# Supplementary material for: Low-Molecular-Weight Collagen Peptide Improves Skin Dehydration and Barrier Dysfunction in Human Dermal Fibrosis Cells and UVB-Exposed SKH-1 Hairless Mice
Source: Int J Mol Sci. 2025 Jul 3;26(13):6427. doi: 10.3390/ijms26136427 (PMC12250359; doi:10.3390/ijms26136427)
Supplement: Supplementary file 1 [file ijms-26-06427-s001.zip › ijms-3720326-supplementary.pdf]

Supplementary Table S1. Western blot antibody.

Target protein	Cat. No.	Dilution	Manufacturer
Phospho-Erk	9101	1: 1000	Cell signaling technology
Erk	9102		
Phospho-JNK	4671		
JNK	9252		
Phospho-p38	4511		
P38	9212		
Phospho-cFos	5348		
cFos	2250		
Phospho-cJun	3270		
cJun	9165		
Beta actin	SC-47778	1: 2500	Santa Curz

Supplementary Table S2. Target mRNA of primer sequences.

Species	Gene	Target sequence (5' - 3')
Mouse	*Mmp-1*	F: AGGAAGGCGATATTGTGCTCTCC
		R: TGGCTGGAAAGTGTGAGCAAGC
	Mmp-2	F: CAAGGATGGACTCCTGGCACAT
		R: TACTCGCCATCAGCGTTCCCAT
	Mmp-9	F: GCTGACTACGATAAGGACGGCA
		R: TAGTGGTGCAGGCAGAGTAGGA
	Mmp-13	F: GATGACCTGTCTGAGGAAGACC
		R: GCATTTCTCGGAGCCTGTCAAC
	Has-1	F: GCTACTTCCACTGTGTGTCCTG
		R: CTAAGCATTCGGTTGGTGAGGTG
	Il-1α	F: CATCTGTGGAGATGGTGAAGGTC

		R: CACTCTGGTAGGTGTAAGGTGC
	Il-1β	F: TGGACCTTCCAGGATGAGGACA
		R: GTTCATCTCGGAGCCTGTAGTG
	Il-6	F: TACCACTTCACAAGTCGGAGGC
		R: CTGCAAGTGCATCATCGTTGTTC
	Il-10	F: CGGGAAGACAATAACTGCACCC
		R: CGGTTAGCAGTATGTTGTCCAGC
	Tnf-α	F: GGTGCCTATGTCTCAGCCTCTT
		R: GCCATAGAACTGATGAGAGGGAG
	Gapdh	F: ACATCATCCCTGCATCCACT
		R: GGGAGTTGCTGTTGAAGTCA
